# Arginase 1 (*Arg1*) as an Up-Regulated Gene in COVID-19 Patients: A Promising Marker in COVID-19 Immunopathy

**DOI:** 10.3390/jcm10051051

**Published:** 2021-03-04

**Authors:** Afshin Derakhshani, Nima Hemmat, Zahra Asadzadeh, Moslem Ghaseminia, Mahdi Abdoli Shadbad, Golamreza Jadideslam, Nicola Silvestris, Vito Racanelli, Behzad Baradaran

**Affiliations:** 1 Immunology Research Center, Tabriz University of Medical Sciences, Tabriz 5165665811, Iran; afshin.derakhshani94@gmail.com (A.D.); nima.hemmat1995@gmail.com (N.H.); Zahraasadzadeh2834@gmail.com (Z.A.); abdoli.med99@gmail.com (M.A.S.); 2 Department of Virology, Faculty of Medicine, Tabriz University of Medical Sciences, Tabriz 5166614766, Iran; moslemghaseminia7386@gmail.com; 3 Student Research Committee, Tabriz University of Medical Sciences, Tabriz 5166614766, Iran; 4 Department of Molecular Medicine, Faculty of Advanced Medical Sciences, Tabriz University of Medical Sciences, Tabriz 5166614766, Iran; gjadideslam@gmail.com; 5 IRCCS Istituto Tumori “Giovanni Paolo II” of Bari, 70124 Bari, Italy; n.silvestris@oncologico.bari.it; 6 Department of Biomedical Sciences and Human Oncology (DIMO), University of Bari, 70124 Bari, Italy; 7 Department of Immunology, Faculty of Medicine, Tabriz University of Medical Sciences, Tabriz 5166614766, Iran

**Keywords:** Arg1, SARS-CoV-2, COVID-19, antiviral immunity, global pandemic

## Abstract

Background: The coronavirus disease 2019 (COVID-19) outbreak, caused by severe acute respiratory syndrome coronavirus 2 (SARS-CoV-2), has been declared a global pandemic. It is well-established that SARS-CoV-2 infection can lead to dysregulated immune responses. Arginase-1 (Arg1), which has a pivotal role in immune cells, can be expressed in most of the myeloid cells, e.g., neutrophils and macrophages. Arg1 has been associated with the suppression of antiviral immune responses. Methods: Whole blood was taken from 21 COVID-19 patients and 21 healthy individuals, and after RNA extraction and complementary DNA (cDNA) synthesis, gene expression of Arg1 was measured by real-time PCR. Results: The qPCR results showed that the expression of *Arg1* was significantly increased in COVID-19 patients compared to healthy individuals (*p* < 0.01). The relative expression analysis demonstrated there were approximately 2.3 times increased *Arg1* expression in the whole blood of COVID-19 patients. Furthermore, the receiver operating characteristic (ROC) analysis showed a considerable diagnostic value for *Arg1* expression in COVID-19 (*p* = 0.0002 and AUC = 0.8401). Conclusion: *Arg1* might be a promising marker in the pathogenesis of the disease, and it could be a valuable diagnostic tool.

## 1. Introduction

The SARS-CoV-2 infection is responsible for the current global pandemic [1]. The rapid spread of SARS-CoV-2 has posed a daunting challenge to healthcare systems worldwide [2].

Although the immune system has a central role in preventing and eliminating viral infections, dysregulated immune responses might be the main reason for the morbidity and the mortality in coronavirus disease 2019 (COVID-19) patients [3,4]. Indeed, the dysregulated immune system can lead to lymphopenia, aberrant stimulation of lymphocytes, neutrophilia, high cytokine levels, and high levels of antibodies [5]. Therefore, immunological biomarkers might be promising biomarkers and therapeutic targets for affected patients [6].

The innate immune system, which recognizes pathogens and microbes and induces proinflammatory cytokines to activate immune responses. Indeed, during the recognition of pathogens, various factors can attract proinflammatory cells, e.g., macrophages and neutrophils, to the infection site to develop inflammatory responses [7]. As the first defense line against pathogens, neutrophils have pivotal roles in inducing inflammation [8]. Although neutrophils can demonstrate antiviral activities in the early stages, they can mediate dysregulated inflammation in coronavirus-induced pneumonia [9,10]. Indeed, neutrophils contribute to the SARS-CoV-2-induced immunopathology in COVID-19 patients [10,11]. This immunopathology is thought to be stemmed from the dysregulated production of cytokines and chemokines that can lead to the “cytokine storm”. The cytokine storm can contribute to acute respiratory distress syndrome, severe inflammatory response syndrome, and sepsis development during COVID-19 [10,12].

It has been reported that the *Arg1* gene is located in the cytoplasm and is strongly expressed in the liver. In addition to its metabolic role in the hepatic urea cycle, it can regulate immune responses. Almost all types of myeloid cells can produce Arg1 [13]. Arg1 can be released to the extracellular microenvironment during inflammatory conditions, e.g., asthma and infectious diseases [14,15,16]. Arg1 inhibits immunity against intracellular pathogens and represses T-cell-mediated inflammatory damage [17,18]. High levels of neutrophil accumulation and systemic circulation result in increased systemic Arg1 activity, which results in depleted systemic arginine. Arginine is a substrate for nitric oxide (NO) production, which can induce antiviral activity against RNA viruses, such as SARS-CoV-2 [19,20].

Our previous investigation has suggested that Arg1 has pivotal roles in SARS-CoV-2 infection and might mediate the inflammation and the hemorrhagic lesions in the infected lungs. Moreover, we have proposed that Arg1 up-regulation might be associated with higher virus load in COVID-19 patients [21]. The current study aims to evaluate the expression of Arg1 as a marker in the whole blood of COVID-19 patients.

## 2. Materials and Methods

### 2.1. Patients and Samples

Twenty-one intubated COVID-19 patients, who had been admitted to the intensive care unit (ICU), were randomly chosen for the study. Twenty-one healthy individuals were enrolled as a control group. All participants received and signed written informed consent. The demographic data were collected through a questionnaire and medical records of patients. This research study was approved by the ethics committee of Tabriz University of Medical Sciences (Ethics Code: IR.TBZMED.REC.1399.008).

### 2.2. The RNA Extraction and Complementary DNA Synthesis

Ten ml of venous blood were collected from patients and healthy volunteers. The RNA extraction from whole blood was carried out by TRIzol reagent (RiboEx). The cDNA synthesis was performed according to the manufacturer’s instructions (Bio FACT, Daejeon, South Korea). The cDNA was stored at −20 °C for real-time PCR analyses.

### 2.3. Real-Time PCR

Real-time PCR was performed to assess the expression of the *Arg1* gene using specific primers. The primer sequences for the *Arg1* were: forward 5′-TGATGTTGACGGACTGGACC-3′ and reverse 5′-ATCTAATCCTGAGAGTAGCCCTGT-3′. Moreover, the glyceraldehyde-3-phosphate dehydrogenase (GAPDH) gene with sequences, forward: 5′-AAGGTGAAGGTCGGAGTCAAC-3′ and reverse: 5′-GGGGTCATTGATGGCAACAA-3′ was used as a housekeeping gene for normalization. Relative gene expression was calculated using the comparative 2^(−∆∆Ct)^ method.

### 2.4. Protein–Protein Interaction (PPI)

The search tool for the retrieval of interacting genes/proteins (STRING) apps of Cytoscape v3.8.1 was utilized to find the most connected genes with Arg1 [22,23]. The cut-off criteria were confidences score ≥ 0.700, maximum interactors = 30, and blood tissue specificity = 2.5.

### 2.5. Statistical Analysis

The results were analyzed using GraphPad Prism v8 (GraphPad Software, San Diego, CA, USA, www.graphpad.com) following the student *t*-test method. The *p*-value below 0.05 was considered significant.

## 3. Results

### 3.1. Population Study

Twenty-one patients with COVID-19 disease (mean age was 58.7 ± 17.5 years, male/female ratio: 10/11) and 21 healthy individuals (mean age was 34 ± 4.6 years, male/female ratio: 9/12) were enrolled for this study. The clinical characteristics of the patients were summarized in Table 1.

### 3.2. Arg1 Expression in the COVID-19 Patients

**Figure 1:**
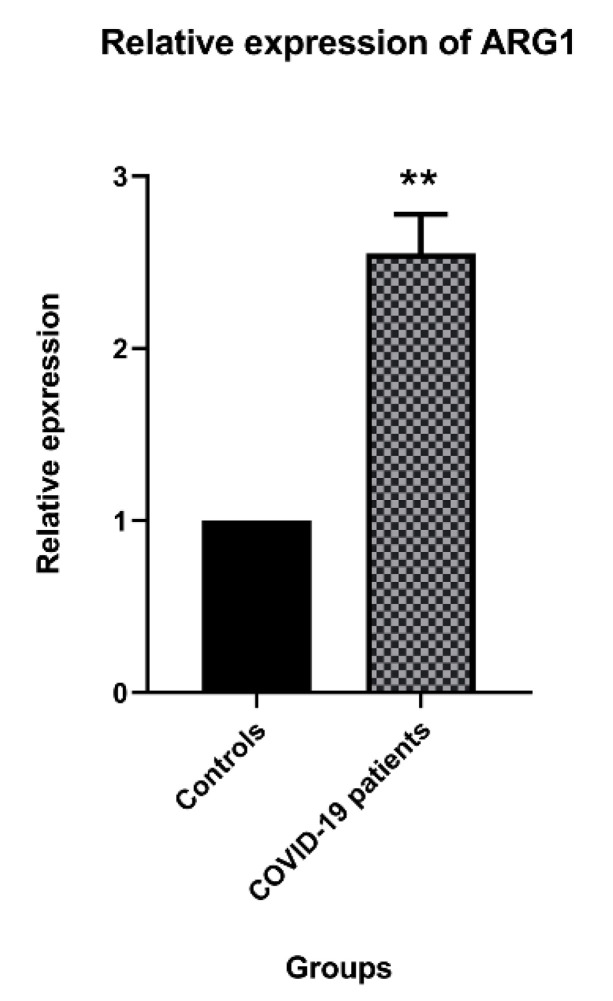
The expression of *Arg1* in coronavirus disease 2019 (COVID-19) patients compared to the control group. The relative expression of *Arg1* in COVID-19 patients was 2.3 times more than in healthy groups. (** *p* < 0.01).

The relative expression analysis demonstrated that *Arg1* was significantly up-regulated in COVID-19 patients compared to healthy individuals (*p* < 0.01) (Figure 1).

### 3.3. The Expression of Arg1 and Underlying Chronic Diseases of COVID-19 Patients

**Figure 2:**
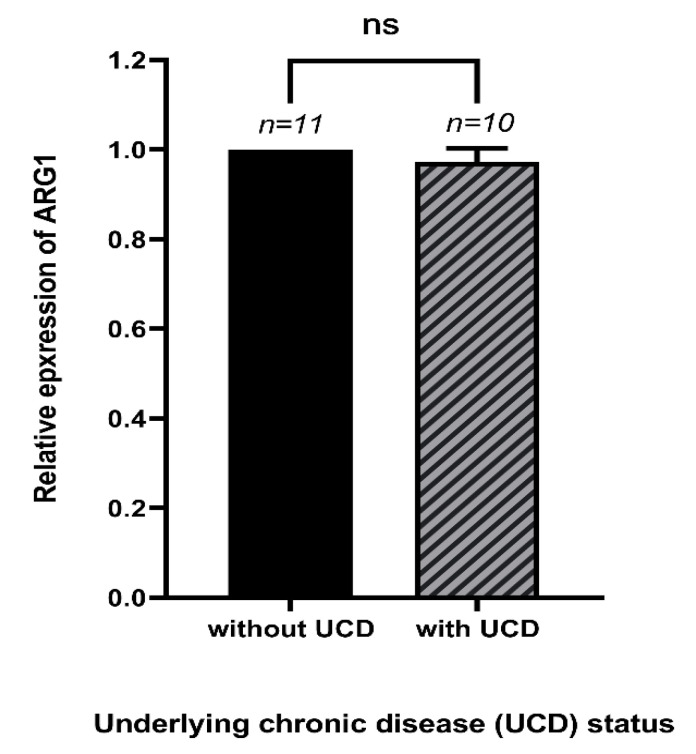
The relative expression of *Arg1* in COVID-19 patients with or without the underlying chronic disease (UCD). There were no significant differences in the expression of *Arg1* between the COVID-19 patients with the UCDs and the COVID-19 patients without UCDs. The numbers of COVID-19 patients with UCDs and without UCDs were 10 and 11, respectively.

Our results showed no significant differences between the expression of *Arg1* in COVID-19 patients with underlying chronic diseases compared to the COVID-19 patients without underlying chronic diseases (*p* > 0.05) (Figure 2). These diseases were chronic heart failure, diabetes, kidney failure, nervous system-related diseases, and hypertension.

### 3.4. The Expression of Arg1 in Dead and Alive COVID-19 Patients

**Figure 3:**
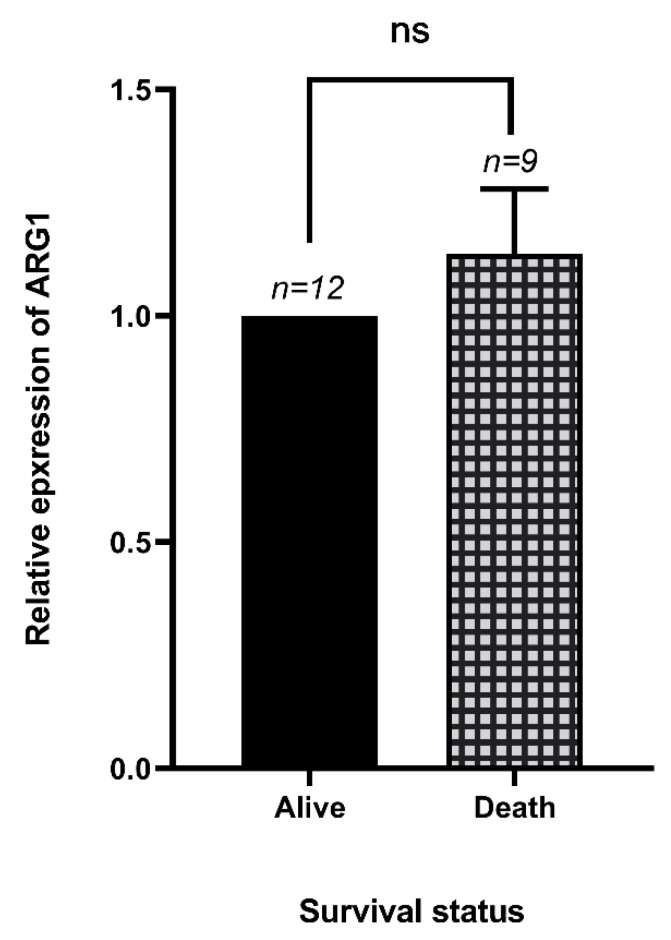
The relative expression of *Arg1* in dead and alive patients of COVID-19. There was an increase in the expression of *Arg1* in dead COVID-19 patients; however, this change was not significant.

Our results showed no significant differences between the *Arg1* expression and the survival status of patients with COVID-19 (*p* > 0.05) (Figure 3).

### 3.5. Arg1 Might Be a Promising Biomarker for COVID-19

**Figure 4:**
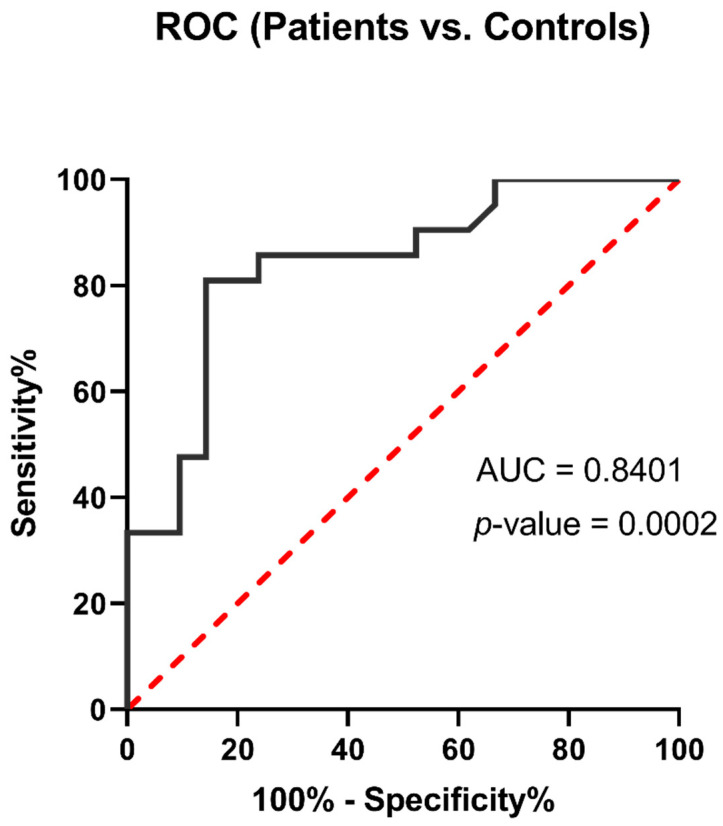
ROC analysis of COVID-19 samples compared to healthy individuals. The analysis showed a significant diagnostic value for *Arg1* gene expression.

The ROC analysis showed a significant diagnostic value for *Arg1* expression in COVID-19 samples compared to control samples (*p* = 0.0002, and AUC = 0.8401) (Figure 4). These results demonstrated that immune activator and genes involved in the enzymatic activity of white blood cells could increase during the COVID-19 and serve as a biomarker.

### 3.6. Arg1 Might Have Interacted with Other Enzymes in the Blood

**Figure 5:**
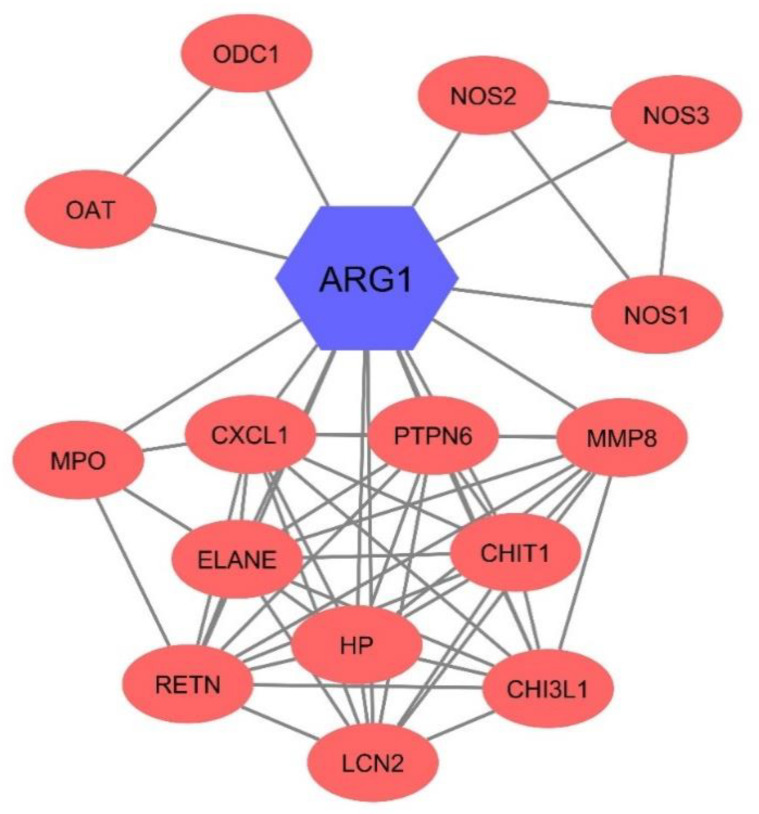
The protein–protein interaction (PPI) network of Arg1. This gene might act as an interactor with several other genes. The PPI enrichment *p*-value of this network is 1.0 × 10^16^.

The PPI network analysis of Arg1 showed that the Arg1 could remarkably interact with ornithine aminotransferase (OAT), nitric oxide synthase 2 (NOS2), and ornithine carbamoyltransferase (OCT) with scores of 0.995, 0.978, and 0.976, respectively (Figure 5). The results confirmed other possible enzymatic changes during the COVID-19 and the involvement of other catalysis enzymes that have considerable associations with Arg1.

## 4. Discussion

Although the immune system is essential for virus elimination, dysregulated immune responses may maintain viral replication. Therefore, a better understanding of SARS-CoV-2-induced immunopathy can reduce the morbidity and the mortality of affected patients [24]. The idea of evaluating *Arg1* in patients with severe COVID-19 came from our previous research in which we analyzed the GSE1739 microarray dataset, including the peripheral blood mononuclear cells (PBMCs) of 10 SARS-positive and the PBMCs of four healthy individuals. Our previous study demonstrated that *Arg1* could be up-regulated in the PBMCs of SARS-CoV-infected patients [21]. Based on the expression level of *Arg1* in the immune cells, e.g., neutrophils, myeloid-derived suppressor cells, monocytes, and macrophages, we aimed to assess the expression of this gene in the whole blood of SARS-CoV2 infected patients to evaluate its potential role in the immune cells and COVID-19-induced immunopathy.

Recent findings have indicated that dysregulated inflammation has a crucial role in the pathogenesis of COVID-19 in patients with severe COVID-19 [25]. Consistent with this, COVID-19 patients demonstrate up-regulated C-reactive protein level, the elevated level of interleukin 6 (IL-6), increased neutrophil counts, and decreased lymphocyte counts [26,27]. Neutrophilia and the elevated level of IL-8 have been associated with worse prognosis in COVID-19 patients [27,28,29]. In the current study, we show that *Arg1* is substantially up-regulated in COVID-19 patients compared to healthy individuals. Moreover, the ROC analysis highlights *Arg1* expression as a valuable diagnostic marker for COVID-19.

Arg1, an essential factor in regulating immune responses, is released from neutrophils during inflammation [26]. Since Arg1 can regulate the bioavailability of L-arginine, it can mediate dysregulated inflammation, the immune evasion of cancer cells, fibrosis, and immunosuppression. L-arginine metabolism, which has complex physiological metabolism, plays a critical role in immune cell reactivity. Indeed, L-arginine, and its downstream metabolites, e.g., ornithine and citrulline, are essential for T-cell stimulation [30,31]. In line with this, Burrack et al. have shown that Arg1 upregulation is associated with elevated viral load and more severe disease in patients with chikungunya virus and Ross River virus. In animal models of the chikungunya virus and the Ross River virus, Arg1 can substantially inhibit antiviral immune responses in affected mice, leading to down-regulated interferon (IFN)-γ expression [12]. Moreover, Santiago-Olivares et al. have shown that constitutive expression of Arg1 might be associated with the maintenance of persistent respiratory syncytial virus infection. They have highlighted that Arg1 inhibition can substantially increase NO and reduce viral genome copy numbers. Since Arg1 can limit the bioavailability of L-arginine, the inhibition of Arg1 can drive the synthesis of NO, paving the way for developing antiviral immunity [32]. Insufficient production of NO may contribute to a defective immune response following infection of mice with an attenuated neurotropic coronavirus (rJ2.2 strain of mouse hepatitis virus). rJ2.2-infected WT mice have exhibited mild acute encephalitis, followed by a non-lethal, chronic demyelinating disease. This defective immune response could also be responsible for the maintenance of viral infection and the induction of the persistence infection phase [33]. In macrophages and neutrophils, specific inhibition of Arg1 can remarkably enhance the clearance of the Ross River virus from musculoskeletal tissues [34]. Although complete inhibition of Arg1 in the lung does not affect the invasion of inflammatory cells, it alters the gene expression profile of these cells in inflammation-induced female mice [35]. Our bioinformatic results have highlighted cross-talk with OAT, NOS2, and OCT. Indeed, macrophage-released NOS2 can convert arginine to nitric oxide and citrulline. The arginase pathway limits the availability of arginine for nitric oxide production, and ornithine, in turn, can further feed into the crucial downstream polyamine and proline synthesis pathways that are vital for wound healing and cell proliferation [36].

One of the most noticeable limitations of our study was the age of the COVID-19 patients was not matched with the age of the healthy individuals. However, our study has several strengths. First, our study showed that the expression of *Arg1* was substantially up-regulated in the whole blood of COVID-19 patients. Furthermore, the *Arg1* level might serve as a valuable diagnostic marker for COVID-19.

## Figures and Tables

**Table 1:** The clinical characteristics of patients.

NO	Sex	Age	UCD	Common Symptoms	LRS	CVS	Other Symptoms	Status
1	Female	68	Heart and kidney failure	Pyrexia and pharyngitis	-	-	-	Dead
2	Male	80	-	Cough	Dyspnea	-	-	Alive
3	Female	89	-	-	Dyspnea	-	-	Dead
4	Male	61	-	Pharyngitis	Dyspnea	-	Muscular pain and loss of appetite	Alive
5	Male	40	Heart failure	Pyrexia	-	-	-	Alive
6	Male	69	Hypertension	Cough	Dyspnea	-	Muscular pain and loss of appetite	Dead
7	Female	56	-	Pyrexia and cough	Dyspnea	-	Hemoptysis	Alive
8	Male	38	Hypertension and diabetes	Pyrexia and cough	Dyspnea	-	Hemoptysis	Dead
9	Female	60	Hypertension	Pyrexia	-	-	Hemoptysis	Alive
10	Female	43	Hypertension	Pyrexia	-	Chest pain	Hemoptysis	Alive
11	Female	62	-	Pharyngitis	Dyspnea	Chest pain	Arthralgia and loss of appetite	Dead
12	Female	64	Hypertension and diabetes	Cough	Dyspnea	Chest pain	Muscular pain, loss of appetite, arthralgia, headache, and vomit	Dead
13	Female	34	-	-	Dyspnea	-	Chills and loss of appetite	Alive
14	Female	45	-	Cough	Dyspnea	-	-	Alive
15	Male	73	Hypertension	Cough	Dyspnea	-	Muscular pain	Alive
16	Male	56	Lung disease	Cough and pharyngitis	Dyspnea	Chest pain	Loss of appetite	Dead
17	Male	88	Nervous and hypertension	-	-	-	-	Alive
18	Female	64	Diabetes and hypertension	Cough	Dyspnea	-	-	Dead
19	Male	81	-	Cough	Dyspnea	-	Loss of appetite	Dead
20	Female	32	-	Cough	Dyspnea	-	Arthralgia and diarrhea	Alive
21	Male	32	-	Pyrexia	Dyspnea	-	-	Alive

Abbreviations: UCD: underlying chronic disease, LRS: lower respiratory symptom, CVS: cardiovascular symptoms.

## Data Availability

Not Applicable.

## References

[1] Maggi E., Canonica G.W., Moretta L. (2020). COVID-19: Unanswered questions on immune response and pathogenesis. J. Allergy Clin. Immunol..

[2] Ling L., Joynt G.M., Lipman J., Constantin J.-M., Joannes-Boyau O. (2020). COVID-19: A critical care perspective informed by lessons learnt from other viral epidemics. Anaesth. Crit. Care Pain Med..

[3] Calder P.C. (2020). Nutrition, immunity and COVID-19. BMJ Nutr. Prev. Health.

[4] Burke H., Freeman A., Cellura D., Stuart B.L., Brendish N.J., Poole S., Borca F., Phan H.T., Sheard N., Williams S. (2020). Inflammatory phenotyping predicts clinical outcome in COVID-19. Respir. Res..

[5] Tang Y., Liu J., Zhang D., Xu Z., Ji J., Wen C. (2020). Cytokine storm in COVID-19: The current evidence and treatment strategies. Front. Immunol..

[6] Yang L., Liu S., Liu J., Zhang Z., Wan X., Huang B., Chen Y., Zhang Y. (2020). COVID-19: Immunopathogenesis and Immunotherapeutics. Signal Transduct. Target. Ther..

[7] Dandekar A.A., Perlman S. (2005). Immunopathogenesis of coronavirus infections: Implications for SARS. Nat. Rev. Immunol..

[8] Rosales C. (2018). Neutrophil: A cell with many roles in inflammation or several cell types?. Front. Physiol..

[9] Haick A.K., Rzepka J.P., Brandon E., Balemba O.B., Miura T.A. (2014). Neutrophils are needed for an effective immune response against pulmonary rat coronavirus infection, but also contribute to pathology. J. Gen. Virol..

[10] Liu Y., Du X., Chen J., Jin Y., Peng L., Wang H.H., Luo M., Chen L., Zhao Y. (2020). Neutrophil-to-lymphocyte ratio as an independent risk factor for mortality in hospitalized patients with COVID-19. J. Infect..

[11] Athanasios D. (2020). COVID-19 Hyperinflammation: What about Neutrophils?. mSphere.

[12] Huang C., Wang Y., Li X., Ren L., Zhao J., Hu Y., Zhang L., Fan G., Xu J., Gu X. (2020). Clinical features of patients infected with 2019 novel coronavirus in Wuhan, China. Lancet.

[13] Grzywa T.M., Sosnowska A., Matryba P., Rydzynska Z., Jasinski M., Nowis D., Golab J. (2020). Myeloid cell-derived Arginase in Cancer immune response. Front. Immunol..

[14] Munder M., Schneider H., Luckner C., Giese T., Langhans C.-D., Fuentes J.M., Kropf P., Mueller I., Kolb A., Modolell M. (2006). Suppression of T-cell functions by human granulocyte arginase. Blood.

[15] Burrack K.S., Tan J.J., McCarthy M.K., Her Z., Berger J.N., Ng L.F., Morrison T.E. (2015). Myeloid cell Arg1 inhibits control of arthritogenic alphavirus infection by suppressing antiviral T cells. PLoS Pathog..

[16] Munder M. (2009). Arginase: An emerging key player in the mammalian immune system. Br. J. Pharmacol..

[17] Zea A.H., Rodriguez P.C., Atkins M.B., Hernandez C., Signoretti S., Zabaleta J., McDermott D., Quiceno D., Youmans A., O’Neill A. (2005). Arginase-producing myeloid suppressor cells in renal cell carcinoma patients: A mechanism of tumor evasion. Cancer Res..

[18] Pesce J.T., Ramalingam T.R., Mentink-Kane M.M., Wilson M.S., El Kasmi K.C., Smith A.M., Thompson R.W., Cheever A.W., Murray P.J., Wynn T.A. (2009). Arginase-1–expressing macrophages suppress Th2 cytokine–driven inflammation and fibrosis. PLoS Pathog..

[19] Caldwell R.W., Rodriguez P.C., Toque H.A., Narayanan S.P., Caldwell R.B. (2018). Arginase: A multifaceted enzyme important in health and disease. Physiol. Rev..

[20] Tatum D., Taghavi S., Houghton A., Stover J., Toraih E., Duchesne J. (2020). Neutrophil-to-lymphocyte ratio and outcomes in Louisiana Covid-19 patients. Shock.

[21] Hemmat N., Derakhshani A., Bannazadeh Baghi H., Silvestris N., Baradaran B., De Summa S. (2020). Neutrophils, Crucial, or Harmful Immune Cells Involved in Coronavirus Infection: A Bioinformatics Study. Front. Genet..

[22] Shannon P., Markiel A., Ozier O., Baliga N.S., Wang J.T., Ramage D., Amin N., Schwikowski B., Ideker T. (2003). Cytoscape: A software environment for integrated models of biomolecular interaction networks. Genome Res..

[23] Doncheva N.T., Morris J.H., Gorodkin J., Jensen L.J. (2018). Cytoscape StringApp: Network analysis and visualization of proteomics data. J. Proteome Res..

[24] García L.F. (2020). Immune response, inflammation, and the clinical spectrum of COVID-19. Front. Immunol..

[25] Lotfinejad P., Asadzadeh Z., Najjary S., Somi M.H., Hajiasgharzadeh K., Mokhtarzadeh A., Derakhshani A., Roshani E., Baradaran B. (2020). COVID-19 Infection: Concise Review Based on the Immunological Perspective. Immunol. Investig..

[26] Xu Z., Shi L., Wang Y., Zhang J., Huang L., Zhang C., Liu S., Zhao P., Liu H., Zhu L. (2020). Pathological findings of COVID-19 associated with acute respiratory distress syndrome. Lancet Respir. Med..

[27] Liu J., Li S., Liu J., Liang B., Wang X., Wang H., Li W., Tong Q., Yi J., Zhao L. (2020). Longitudinal characteristics of lymphocyte responses and cytokine profiles in the peripheral blood of SARS-CoV-2 infected patients. EBioMedicine.

[28] Mez J., Daneshvar D.H., Kiernan P.T., Abdolmohammadi B., Alvarez V.E., Huber B.R., Alosco M.L., Solomon T.M., Nowinski C.J., McHale L. (2017). Clinicopathological evaluation of chronic traumatic encephalopathy in players of American football. JAMA.

[29] Zhang X., Tan Y., Ling Y., Lu G., Liu F., Yi Z., Jia X., Wu M., Shi B., Xu S. (2020). Viral and host factors related to the clinical outcome of COVID-19. Nature.

[30] Li Z., Zhao Z.-J., Zhu X.-Q., Ren Q.-S., Nie F.-F., Gao J.-M., Gao X.-J., Yang T.-B., Zhou W.-L., Shen J.-L. (2012). Differences in iNOS and arginase expression and activity in the macrophages of rats are responsible for the resistance against T. gondii infection. PLoS ONE.

[31] Peranzoni E., Marigo I., Dolcetti L., Ugel S., Sonda N., Taschin E., Mantelli B., Bronte V., Zanovello P. (2008). Role of arginine metabolism in immunity and immunopathology. Immunobiology.

[32] Santiago-Olivares C., Rivera-Toledo E., Gómez B. (2019). Nitric oxide production is downregulated during respiratory syncytial virus persistence by constitutive expression of arginase 1. Arch. Virol..

[33] Burrack K.S., Morrison T.E. (2014). The role of myeloid cell activation and arginine metabolism in the pathogenesis of virus-induced diseases. Front. Immunol..

[34] Stoermer K.A., Burrack A., Oko L., Montgomery S.A., Borst L.B., Gill R.G., Morrison T.E. (2012). Genetic ablation of arginase 1 in macrophages and neutrophils enhances clearance of an arthritogenic alphavirus. J. Immunol..

[35] Cloots R.H., Sankaranarayanan S., Poynter M.E., Terwindt E., van Dijk P., Lamers W.H., Köhler S.E. (2017). Arginase 1 deletion in myeloid cells affects the inflammatory response in allergic asthma, but not lung mechanics, in female mice. BMC Pulm. Med..

[36] Rath M., Müller I., Kropf P., Closs E.I., Munder M. (2014). Metabolism via arginase or nitric oxide synthase: Two competing arginine pathways in macrophages. Front. Immunol..

